# Creating a hierarchy of mental health stigma: testing the effect of psychiatric diagnosis on stigma

**DOI:** 10.1192/bjo.2022.578

**Published:** 2022-09-26

**Authors:** Cassie M. Hazell, Clio Berry, Leanne Bogen-Johnston, Moitree Banerjee

**Affiliations:** School of Social Sciences, University of Westminster, UK; Department of Primary Care and Public Health, Brighton and Sussex Medical School, University of Sussex and University of Brighton, UK; and School of Psychology, University of Sussex, UK; Research & Development Department, Sussex Partnership NHS Foundation Trust, UK; and School of Psychology, University of Sussex, UK; Institute of Education, Social and Life Sciences, University of Chichester, UK

**Keywords:** Stigma and discrimination, education and training, phenomenology, rating scales, community mental health teams

## Abstract

**Background:**

Levels of mental health stigma experienced can vary as a function of the presenting mental health problem (e.g. diagnosis and symptoms). However, these studies are limited because they exclusively use pairwise comparisons. A more comprehensive examination of diagnosis-specific stigma is needed.

**Aims:**

The aim of our study was to determine how levels of mental health stigma vary in relation to a number of psychiatric diagnoses, and identify what attributions predict levels of diagnosis-specific stigma.

**Method:**

We conducted an online survey with members of the public. Participants were assessed in terms of how much stigma they had, and their attributions toward, nine different case vignettes, each describing a different mental health diagnosis.

**Results:**

We recruited 665 participants. After controlling for social desirability bias and key demographic variables, we found that mental health stigma varied in relation to psychiatric diagnosis. Schizophrenia and antisocial personality disorder were the most stigmatised diagnoses, and depression, generalised anxiety disorder and obsessive–compulsive disorder were the least stigmatised diagnoses. No single attribution predicted stigma across diagnoses, but fear was the most consistent predictor.

**Conclusions:**

Assessing mental health stigma as a single concept masks significant between-diagnosis variability. Anti-stigma campaigns are likely to be most successful if they target fearful attributions.

Mental health stigma can be understood as the combination of a lack of knowledge (ignorance), negative attitudes (prejudice) and disadvantaging behaviour (discrimination) toward those with mental health problems.^1^ The effects of mental health stigma are pervasive: stigma reduces help-seeking,^2,3^ has adverse economic implications via increased unemployment and healthcare costs,^4^ and worsens both the mental^5^ and physical health^6^ of the stigmatised individual. Although mental health stigma may have improved over time,^7,8^ current levels are still problematic.^9^

Research that has studied mental health stigma as a singular construct (i.e. stigma toward persons with mental health problems generally)^7^ has been criticised for neglecting the heterogeneity of mental health diagnoses. The studies that have addressed this issue have largely compared two diagnoses at a time: one that is a common mental health problem (CMHP) (i.e. most prevalent diagnoses, such as depression and anxiety) and one that is a serious mental illness (SMI) diagnosis (i.e. diagnoses that are thought to be the most debilitating, such as psychosis spectrum disorders and personality disorders). Studies have consistently shown that stigma is greater toward those with an SMI diagnosis than those with a CMHP condition.^10–14^

According to the cognitive–behavioural model of stigma, these differences are the result of differing attitudes.^15^ Specifically, mental health stigma is a product of decreased pity, increased anger and fear, and believing those with mental health problems are personally responsible for their symptoms.^16^ Stigma is therefore theorised to be greater toward those with SMI diagnoses than CMHP because the public perceives those with SMI diagnoses as more dangerous^17^ and have less ‘pity’ for them,^12^ compared with people with a CMHP.

The wealth of studies using pairwise comparisons to investigate diagnosis-specific stigma provide a more nuanced understanding of mental health stigma, but they are limited in their scope. One study that went beyond using a pairwise comparison to investigate stigma also found that out of the four mental health diagnoses/symptoms studied, an SMI condition (i.e., schizophrenia) was the most stigmatised.^18^ This study further confirms diagnosis-related heterogeneity in self-reported stigma, but still does not allow us to draw any conclusions about within-class diagnostic differences. That is, we have little understanding of how self-reported stigma varies among the different CMHPS and various SMI diagnoses. The present study aims to fill this gap in the literature by investigating how self-reported stigma varies in relation to a greater array of diagnoses.

The differences in diagnosis-related stigma has been described in terms of a hierarchy by persons with lived experience of mental health difficulties.^19^ This analogy of a hierarchy does not mean that any mental health problem is more serious or severe than another. Instead, the hierarchy here refers to the grading of mental health stigma in that some mental health diagnoses are discriminated against more frequently and more harshly than others.^20^ The present study will address the aforementioned gap in the literature by using the concept of a mental health stigma hierarchy.

To our knowledge, there has been no study comparing stigma toward such a comprehensive array of SMI and CMHP diagnoses that also tests what attitudes may explain this diagnosis-specific stigma. The findings cultivated here can inform anti-stigma campaigns and initiatives at the public health and organisation level, so that the most stigmatised diagnoses are targeted with the mechanisms (i.e. attitudes) that are most likely to bring about change. The aim of the present study was to first develop a hierarchy of mental health stigma – that is, identify which diagnoses are the most and least stigmatised. Second, we aimed to identify diagnosis-specific predictors of mental health stigma.

## Research questions

The following research questions will be explored:

- Does stigma vary in relation to psychiatric diagnoses?
- How does stigma vary in relation to psychiatric diagnoses?
- Do attributions explain diagnostic differences in mental health stigma?

## Method

### Design

The present study used a repeated-measures, cross-sectional survey design. To investigate whether and how stigma varies in relation to psychiatric diagnoses, the independent variable was the psychiatric diagnosis of one of the nine case vignettes. To reduce bias, the order in which the case vignettes were presented was randomised. To assess whether attributions could explain diagnostic differences in mental health stigma, a model was developed to test if four attributions, measured with the Attribution Questionnaire^16^ subscales, predicted mental health stigma as measured with the Social Distance Scale (SDS).^21^

### Participants

We wanted to assess mental health stigma among the public. To be eligible to participate, persons were required to be aged 18 years or over, and living in the UK. Our power analysis for a repeated-measures analysis of covariance study with nine groups aiming to detect a small effect size (Cohen's *f* = 0.10) indicated a minimum target sample size of 128. Participants were recruited online via social media and survey sharing sites. Specifically, the survey promotional materials were posted on public forums and discussion groups that were both related and unrelated to mental health. Participants were offered the opportunity to enter a prize draw for £50 in exchange for their participation.

### Case vignettes

Case vignettes are text only, and used here to provide descriptive exemplars of particular mental health problems. The first line of each case vignette detailed the patient's diagnosis and then described the core symptoms. We produced case vignettes reflecting nine different psychiatric diagnoses: schizophrenia, bipolar disorder type 1, depression, generalised anxiety disorder (GAD), obsessive–compulsive disorder (OCD), dissociative identity disorder (DID), post-traumatic stress disorder (PTSD), borderline personality disorder (BPD), and antisocial personality disorder (ASPD) (see Supplementary Material available at https://doi.org/10.1192/bjo.2022.578). The case vignettes were based upon psychiatry case examples^22^ that were modified to make them gender neutral and ensure equivalent word lengths. The diagnoses selected are all conditions that are usually treated within standard primary and/or secondary mental health services (i.e. we excluded those diagnoses usually seen in specialist and/or tertiary services, such as substance disorders and dementias). These nine diagnoses cover the majority of the subcategories within the ICD-10 mental health behavioural disorder classifications:^23^ F20–F29 schizophrenia, schizotypal and delusional disorders; F30–F39 mood (affective) disorders; F40–F48 neurotic, stress-related and somatoform disorders; F50–F59 behavioural syndromes associated with physiological disturbances and physical factors; and F60–F69 disorders of adult personality and behaviour. The vignettes used in the present study are available in the Supplementary Material.

### Measures

#### SDS

The SDS measures the extent to which a person wishes to distance themselves from a specific group. In this instance, we used the Link et al^21^ version of the SDS to capture desired distance from each person described in the case vignettes. The SDS has seven items, and has strong internal consistency evidenced in both the original study (*α* = 0.92)^21^ and the present study (all *α* ≥ 0.88). A higher score indicates a greater unwillingness to be close to those with mental health problems, i.e. more stigma.

#### Vignette-specific Attribution Questionnaire

The Attribution Questionnaire^16^ measures various attributions toward each person described in the case vignettes. The questionnaire has 13 items, divided into four subscales: personal responsibility, pity, anger and fear. We found each of these scales had good internal consistency (personal responsibility: *α*s ≥ 0.78; pity: *α*s ≥ 0.85; anger: *α*s ≥ 0.88; fear: *α*s ≥ 0.94). A higher score reflects increased fear, anger and pity toward the person in the case vignette, and an increased belief that their problems are their own fault.

#### Brief Marlowe–Crowne Social Desirability Scale

The Brief Marlowe–Crowne Social Desirability Scale (MCSDS)^24^ assesses the extent to which a participant is susceptible to the social desirability bias. Those susceptible to this bias are more likely to provide favourable responses that differ from their true opinion, and therefore need to be controlled for.^25^ The Brief MCSDS has ten items. We found the scale to have acceptable internal consistency (*α* = 0.69). A higher score indicates greater susceptibility to social desirability.

### Procedure

The survey was promoted online via social media and survey sharing sites, using promotional materials that contained a URL and QR code that linked directly to a participant information statement. After reading this statement, participants were asked to complete an eligibility assessment and provide consent using an online consent form. Written informed consent was obtained from all participants via an online tick box form. After providing demographic information, participants were asked to complete the Brief MCSDS, followed by the SDS and Attribution Questionnaire in relation to each of the nine case vignettes. Finally, participants were presented with a debrief statement and given the opportunity to enter a £50 cash prize draw.

### Ethics

Participants completed the survey anonymously. The authors assert that all procedures contributing to this work comply with the ethical standards of the relevant national and institutional committees on human experimentation and with the Helsinki Declaration of 1975, as revised in 2008. All procedures involving human patients were approved by the University of Westminster Psychology Ethics Committee (application identifier: ETH1920-0344).

### Analysis plan

To assess differences in diagnosis-specific self-reported stigma, we conducted a one-way repeated-measures analysis of covariance. We compared levels of stigma (as measured by the SDS) between the nine psychiatric diagnoses, controlling for susceptibility to social desirability (as measured by the Brief MCSDS), age and dummy variables for gender, ethnicity and lived experience of mental health problems. We used *post hoc* comparisons with a Bonferroni correction to assess differences between all nine diagnoses. The estimated marginal means (EMM) were used to assess the direction of any significant differences. For each case vignette, we tested the theoretical model of attributions as predictors for mental health stigma. The theoretical path analytic model was tested with maximum likelihood parameter estimation, using several fit indices: the Satorra–Bentler chi-squared test, root mean square error of approximation and comparative fit index. A root mean square error of approximation value of less than 0.08 indicates an adequate fit, whereas the comparative fit index ranges from 0 (absolute lack of fit) to 1 (perfect fit). The standardised path coefficients were reported.

## Results

### Missing data

The survey received a total of 834 visits; 24 people did not meet the eligibility criteria (remaining *n* = 810), 82 people did not provide consent (*n* = 728) and seven people provided no data after the consent form (*n* = 721). Of the 721 participants who provided some data, 56 completed the demographic questions only, leaving a final sample of 665.

To test whether there were any patterns regarding data missingness, we conducted a logistic regression testing whether gender (male versus all others, female versus all others and other gender identity versus all others), age or ethnicity (White versus all other ethnicities) predicted data missingness (demographic data only versus complete data). Only the dummy variables for male (*b* = 4.84, *P* < 0.001) and female (*b* = 5.14, *P* < 0.001) were significant, whereby data missingness was predicted by being male versus all other genders, or female versus all other genders. All other characteristics were non-significant (all *p* ≥ 0.09).

### Sample characteristics

The characteristics of our sample are reported in Table 1. Our sample had an average age of 33.42 years, and were largely female, White British, in paid employment, single, with an educational qualification and no reported disability. The majority of our sample did not have any personal experience of poor mental health or a mental health crisis.

**Table 1:** Sample characteristics

	*n*	*n* (%) or mean (s.d.)
Demographics		
Age	665	33.42 (12.30)
Gender	665	
Male		190 (28.6)
Female		471 (70.8)
Other		3 (0.5)
Prefer not to say		1 (0.2)
Ethnicity	663	
White British		334 (50.4)
White other		62 (9.4)
Chinese/Chinese British		13 (2.0)
Black/African/Caribbean/Black British		38 (5.7)
Asian/Asian British		186 (28.1)
Mixed ethnicity		20 (3.0)
Other		3 (0.5)
Prefer not to say		7 (1.1)
Employment status	660	
Employed paid (full or part time)		300 (45.4)
Employed voluntary (full or part time)		22 (3.4)
Unemployed		48 (7.3)
Student		192 (29.1)
Retired		16 (2.4)
Self-employed		38 (5.8)
Homemaker		22 (3.3)
Other		17 (2.6)
Prefer not to say		5 (0.8)
Marital status	663	
Single		285 (43.0)
Married/civil partnership		206 (31.1)
Cohabiting		119 (17.9)
Separated/divorced		31 (4.7)
Widowed		7 (1.1)
Prefer not to say		15 (2.3)
Educational qualification	664	
No formal qualification		21 (3.2)
Secondary/high school qualification		107 (16.1)
College/sixth form qualification		207 (31.2)
Undergraduate degree or equivalent		204 (30.7)
Postgraduate degree or equivalent		87 (13.1)
Other		30 (4.5)
Prefer not to say		8 (1.2)
Disability	664	
Yes		119 (17.9)
No		512 (77.1)
Prefer not to say		33 (5.0)
Mental health characteristics		
Personal experience	663	
Yes, and I have a diagnosis		167 (25.2)
Yes, but I do not have a diagnosis		93 (14.0)
No		403 (60.8)
Experience of mental health crisis	664	
Yes, and I was hospitalised		66 (9.9)
Yes, but I was not hospitalised		78 (11.7)
No		520 (78.3)

Disability status is defined as any condition that meets the criteria for a disability under the Equality Act 2010; a mental health crisis is defined as any incident where emergency mental health support was needed (e.g. ambulance, police, mental health rapid response team).

### Interaction with covariates

There was a significant interaction between the case vignette diagnosis and participant ethnicity (*F*(6.01, 3056.34) = 7.96, *P* < 0.001), social desirability (*F*(6.01, 3056.34) = 40.97, *P* < 0.001), age (*F*(6.01, 3056.34) = 2.50, *P* = 0.02) and lived experience of mental health (*F*(6.01, 3056.34) = 14.91, *P* < 0.001). All other covariate interactions were non-significant (*p*s > 0.05).

### Does stigma vary in relation to different psychiatric diagnosis?

The assumption of sphericity was not met (Mauchly's *W* = 0.37, *χ^2^*(35) = 499.21, *P* < 0.001) – we therefore used a Greenhouse–Geisser correction. After controlling for susceptibility to social desirability bias, demographic characteristics and lived experience of mental health problems, the main effect of diagnosis on stigma was non-significant (*F*(6.01, 3056.34) = 0.56, *P* = 0.76).

### How does stigma vary in relation to specific psychiatric diagnoses?

The results of the *post hoc* pairwise comparisons are reported in Table 2. The results show that there was no significant difference in stigma toward depression versus GAD (*P* = 0.34), GAD versus OCD (*P* = 0.06), BPD versus ASPD (*P* = 1.00) and ASPD versus schizophrenia (*P* = 0.10). All other pairwise comparisons were significant (*P* < 0.001), meaning that participants self-reported stigma levels varied in relation to these diagnoses.

**Table 2:** Mean difference, exact *P*-values and statistical significance of pairwise comparisons

	Case vignette diagnosis								
	Schizophrenia	DID	Depression	GAD	Bipolar disorder type 1	OCD	PTSD	BPD	ASPD
	A	B	C	D	E	F	G	H	I
A	−	−0.30 (0.000^a^)	−1.18 (0.000^a^)	−1.12 (0.000^a^)	−0.52 (0.000^a^)	−1.05 (0.000^a^)	−0.62 (0.000^a^)	−0.13 (0.000^a^)	−0.08 (0.10)
B		−	−0.88 (0.000^a^)	−0.82 (0.000^a^)	−0.22 (0.000^a^)	−0.75 (0.000^a^)	−0.32 (0.000^a^)	0.17 (0.000^a^)	0.22 (0.000^a^)
C			−	0.06 (0.34)	0.67 (0.000^a^)	0.13 (0.000^a^)	0.56 (0.000^a^)	1.05 (0.000^a^)	1.10 (0.000^a^)
D				−	0.60 (0.000^a^)	0.07 (0.06)	0.50 (0.000^a^)	0.99 (0.000^a^)	1.04 (0.000^a^)
E					−	−0.53 (0.000^a^)	−0.10 (0.001^a^)	0.39 (0.000^a^)	0.44 (0.000^a^)
F						−	0.43 (0.000^a^)	0.92 (0.000^a^)	0.96 (0.000^a^)
G							−	0.49 (0.000^a^)	0.63 (0.000^a^)
H								−	0.05 (1.00)
I									−

Numbers in parentheses are *P-*values. DID, dissociative identity disorder; GAD, generalised anxiety disorder; OCD, obsessive–compulsive disorder; PTSD, post-traumatic stress disorder; BPD, borderline personality disorder; ASPD, antisocial personality disorder.

a. Statistically significant comparison.

**Fig. 1:**
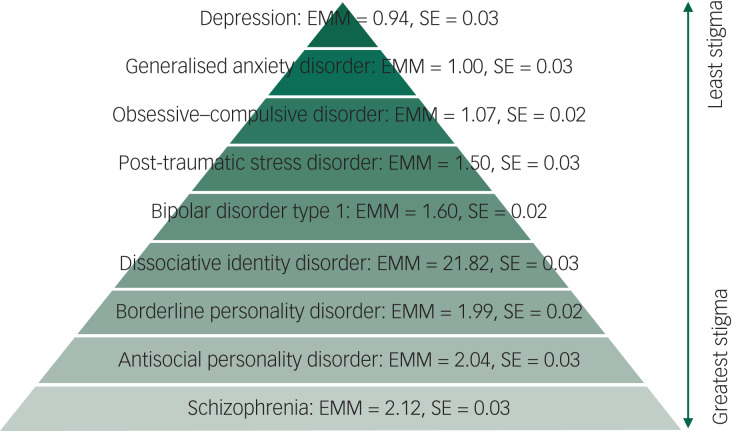
The hierarchy of mental health stigma. EMM, estimated marginal means.

Observation of the EMMs shows that stigma was lowest for depression, GAD and OCD, and highest for BPD, ASPD and schizophrenia. In order from lowest to highest, the stigma hierarchy was as follows: depression, GAD, OCD, PTSD, bipolar disorder type 1, DID, BPD, ASPD and schizophrenia. The hierarchy of stigma, according to psychiatric diagnoses, with descriptive statistics is reported in Figure 1.

### Do attributions explain differences in stigma toward psychiatric diagnoses?

**Fig. 2:**
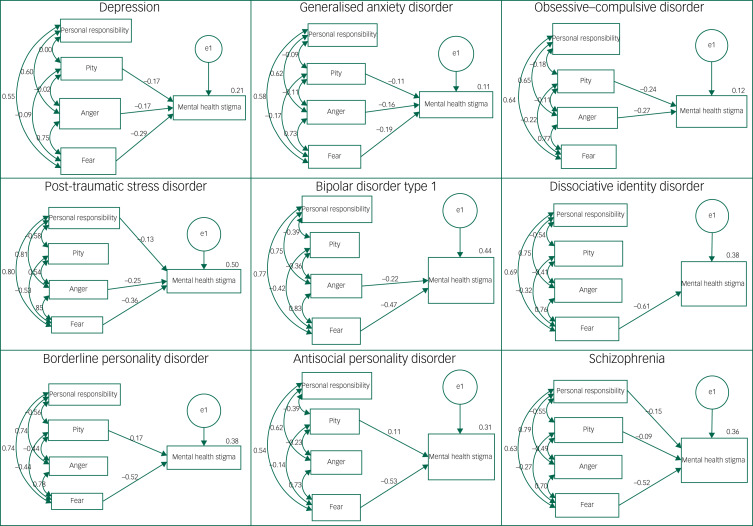
Path analytic models with standardised coefficients for each mental health diagnosis. Models show only significant path coefficients (*P* < 0.01); double-headed arrows depict covariance.

The path analytic model was tested separately for each diagnosis. The overall fit indices for each predictive model, as well as the size and significance of individual predictors, are reported in Table 3 and Figure 2. The attribution that most consistently predicted stigma across diagnoses was fear; whereby greater perceived fear in relation to the diagnosis was associated with increased stigma for all diagnoses except OCD. By contrast, increased perceptions of personal responsibility predicted more stigma for only schizophrenia and PTSD. Higher levels of mental health stigma were predicted by increased pity for case vignettes describing someone with depression, GAD, OCD, BPD and schizophrenia, and decreased pity toward ASPD. Finally, increased anger associated with the case vignette predicted increased stigma with regard to depression, GAD, OCD, PTSD, bipolar disorder type 1 and BPD diagnoses.

**Table 3:** Fit indices for the derived path models in relation to diagnoses

	Fit indices			
	RMSEA (90% CI)	*χ*^2^ (d.f.)	*χ^2^*/d.f.	CFI
Schizophrenia	0.15 (0.00–0.10)	1.17 (1)	1.17	**1**.**00**
Depression	**0.03** (0.00–0.11)	1.67 (1)	1.67	**0**.**99**
GAD	**0.00** (0.00–0.08)	0.32 (1)	0.32	**1**.**00**
OCD	**0.00** (0.00–0.06)	0.83 (2)	0.42	**1**.**00**
PTSD	**0.00** (0.00–0.04)	0.01 (1)	0.01	**1**.**00**
Bipolar disorder type 1	**0.00** (0.00–0.08)	0.40 (1)	0.40	**1**.**00**
DID	**0.00** (0.00–0.04)	0.81 (3)	0.27	**1**.**00**
BPD	**0.04** (0.00–0.11)	1.86	1.86	**0**.**99**
ASPD	**0.00** (0.00–0.07)	1.49 (2)	0.75	**0**.**99**

Bold indices indicate good model fit; RMSEA <0.08 and CFI >0.9 indicate adequate fit. RMSEA, root mean square error of approximation; CFI, comparative fit index; GAD, generalised anxiety disorder; OCD, obsessive–compulsive disorder; PTSD, post-traumatic stress disorder; DID, dissociative identity disorder; BPD, borderline personality disorder; ASPD, antisocial personality disorder.

## Discussion

The aim of the present study was to develop a hierarchy of mental health stigma and test what attributions predict diagnosis-specific stigma. We conducted an online survey in which participants rated their stigma and attributions toward case vignettes describing nine persons, each with a different psychiatric diagnosis. We found that the diagnoses attracting the most stigma were schizophrenia and ASPD, whereas depression, GAD and OCD were the least stigmatised. There was significant variation in the extent to which attributions predicted mental health stigma across diagnoses. Believing that the person with mental health problems was personally responsible for their symptoms only predicted stigma for a minority of diagnoses, whereas being fearful of the diagnosis predicted stigma for most diagnoses.

The hierarchy of mental health stigma established here broadly mirrors a CMHP versus SMI distinction. That is, mental health stigma was lower for CMHP compared with SMI diagnoses. This aligns with previous studies that have conducted pairwise comparisons of diagnosis-specific mental health stigma,^10–14^ whereby CMHP diagnoses were less stigmatised than all SMI diagnoses. The CMHP/SMI distinction found here may be explained by the differing prevalence of these disorders. Diagnoses that are more common are likely to breed greater familiarity, and familiarity is associated with reduced stigma.^26^ The diagnoses in the middle of the hierarchy are those that bridge the CMHP/SMI divide in terms of their prevalence and presentation. Diagnoses such as PTSD and bipolar disorder present with symptoms associated with CMHP, such as low mood and anxiety, but can also present with less common, SMI symptoms, such as psychosis and dissociation. The hierarchy therefore reflects the decreasing commonality of the diagnoses, and the increasing probability of the patient presenting with unusual symptoms.

The descriptive data shows that schizophrenia was the most stigmatised diagnosis. This finding compliments extensive research demonstrating that people with psychosis frequently encounter public stigma,^27–29^ as do their family and friends.^30,31^ Such experiences of stigma increase isolation, limit access to social and employment opportunities,^27^ worsen symptoms^32^ and impede help-seeking and treatment efficacy.^27^ The results of statistical tests, however, found no significant difference between stigma in relation to schizophrenia and ASPD. Similarly, the stigma experienced by people with ASPD can negatively affect their mental health and treatment experiences.^33^

The stigma associated with both schizophrenia and ASPD was predicted by increased fear. The diagnostic criteria for ASPD specifically highlights symptoms/characteristics associated with harmful behaviours (e.g. deceitfulness, callousness and manipulativeness),^23–34^ and is highly prevalent within forensic services and the criminal justice system.^35^ It could therefore be argued that fears toward ASPD have some legitimacy. This position is tenuous, however, when applied to schizophrenia, as individuals with psychosis are more likely to be victims of crime than perpetrators.^36,37^ Despite differences in the likelihood of threat associated with each diagnosis, we found no difference in the levels of mental health stigma. This can be attributed to the mainstream media's portrayal of people with schizophrenia as dangerous and violent.^38^ We can therefore conclude that the public do not necessarily form attributions about mental health problems based on accurate information.

Attitudes are a core component of the cognitive–behavioural model of mental health stigma.^15^ The findings here suggest that the cognitive–behavioural model of mental health stigma must be tailored to the given diagnosis, as attributions found to predict mental health stigma varied by diagnosis. For example, pity did not predict mental health stigma toward people with PTSD, bipolar disorder and DID, but was a significant predictor in all other path models. Pity is therefore not a reliable target for reducing mental health stigma generally.

### Limitations

Our sample was predominantly White, female, employed and educated – this modal participant profile is associated with less stigma toward people with mental health problems generally.^9^ We tried to mitigate this limitation by controlling for key demographics in the analysis, but the overall stigma scores found here may still be an underestimation, and may not completely generalisable to the wider UK population. Also, we may have inadvertently assessed self-stigma for a proportion of our participants. A quarter of our sample had a diagnosed mental health problem that may have been one of the diagnoses described in a case vignette. We included lived experience as a covariate, but we cannot further explore the presence of self-stigma as we did not ask participants for their diagnosis. Self-report measures of mental health stigma can be susceptible to social desirability biases.^39^ We tried to address this by using an online survey, which is considered to be the data collection method that offers the greatest anonymity and therefore the least bias;^25^ and controlling for social desirability in our analysis by using the Brief MCSDS,^24^ i.e. testing the effect of diagnosis on stigma after removing the impact of social desirability. However, in some instances these measures were not sufficient to control for the influence of social desirability.^40^ Despite this limitation, we still found several significant differences in diagnosis-related stigma. It is therefore possible that if we used implicit measures of stigma, where the impact of social desirability is negligible, that the between-diagnosis differences may be even greater – this requires testing in a future research study.

We used case vignettes to test diagnosis-specific differences in mental health stigma. The first line of each case vignette stated the patient's diagnosis, followed by a summary of key symptoms. We therefore cannot disentangle the impact of the diagnostic label versus symptom description on the stigma reported. It is possible that the differences found here would be reduced if we removed the diagnostic labels, as the general public are not able to consistently identify psychiatric diagnoses from descriptions of presenting symptoms,^41^ and that diagnostic labelling increases stigma.^11^ This hypothesis requires verification, but could provide a compelling argument against the use of diagnosis in mainstream mental health services. Also, these vignettes were based on case examples given in a psychiatry training textbook;^22^ however, mental health difficulties are heterogeneous with differing combinations and prominence of presenting symptoms that may have different perceived severities.^42^ Our vignettes describe the most prototypical presentation of each of the mental health problems, but it may be that if other symptoms were included and/or brought to the fore in the vignettes, therein changing the perceived severity of the mental health difficulty, our findings could have differed.

### Research implications

The hierarchy established here requires replication. Future studies in this area should seek to recruit participants that are representative of the public. We also need to confirm whether this hierarchy is in line with the perspectives of people with lived experience and mental health professionals. Beyond this, other research priorities include replicating our study with implicit measures of mental health stigma and case vignettes without the diagnoses. These studies will address limitations related to social desirability and the impact of diagnosis on stigma. We measured mental health stigma in terms of social distance^21^ and assessed attitudinal predictors.^16^ There are other aspects of mental health stigma not considered in our model here that require consideration in future studies (i.e. ignorance). Additionally, there are other variables not explored here that could be important in predicting mental health stigma. For example, mental health literacy^43^ has been found to predict levels of mental health stigma. Future research should consider how best to reduce fear-related attributions as a means of reducing stigma, especially in the context of schizophrenia.

### Clinical implications

The purpose of establishing a hierarchy of mental health stigma is to evidence that although people with mental health problems generally do experience stigma, this stigma is not equivalent across diagnoses and the correlates of this stigma may also be diagnosis specific. Continued efforts are needed to address mental health stigma broadly, but our findings suggest that anti-stigma campaigns need to particularly feature SMI conditions, as they are the most stigmatised. These anti-stigma campaigns are likely to be most effective if they take a diagnosis-specific approach, based on our regression models and the attributions found here to significantly predict stigma. The application of this hierarchy in the real world would benefit from input from those with lived experience to avoid its misuse in terms of prioritising the impact of stigma on someone with a particular diagnosis over another.

## Data Availability

The data that support the findings of this study are available from the corresponding author, C.M.H., upon reasonable request.

## Supplementary material

For supplementary material accompanying this paper visit http://doi.org/10.1192/bjo.2022.578. click here to view supplementary material
