# Supplementary material for: Creating a hierarchy of mental health stigma: testing the effect of psychiatric diagnosis on stigma
Source: BJPsych Open. 2022 Sep 26;8(5):e174. doi: 10.1192/bjo.2022.578 (PMC9534883; doi:10.1192/bjo.2022.578)
Supplement: Supplementary file 1 [file bjosup.zip › S2056472422005786sup001.docx]

Supplementary Material.

# Schizophrenia spectrum:

Patient A has a diagnosis of Schizophrenia. Patient A appears suspicious and guarded, avoiding eye contact. When interviewed, the patient declines to answer any questions because “they are listening to us”. The patient won’t provide any of their personal information because “they can’t know where I am. They are going to hurt me”. It is difficult to follow their speech and they often turn their head away and mumble to themselves. They look around abruptly several times and appear to be responding to something that others can’t see or hear.

# Bipolar Type I:

Patient E has a diagnosis of Bipolar Type I. Patient E has been working solidly for the last 4 days on a project that will solve the climate crisis. Their family has tried to convince them to take a break, but they reject this advice accusing them of being on “their side”. The patient will talk to everyone about the project – their enthusiasm is palpable. They haven’t had much sleep lately, but they still have loads of energy. When they talk about their project it is difficult to follow their thought process.

# Depression:

Patient C has a diagnosis of Depression. Patient C reports feeling really low in mood. They don’t want to go out and socialise – they just want to be left alone. They have had problems getting off to sleep and wake up several times in the night; they also feel hungrier than usual. They tried to read the newspaper but had to keep reading the same line over and over again before they understood what it was about. The patient’s favourite hobby was painting, but now painting feels like a chore.

# Generalised Anxiety Disorder:

Patient D has a diagnosis of Generalised Anxiety Disorder. Patient D is finding it hard to concentrate and keeps getting distracted by multiple worries. They are really restless, and keep fidgeting with their bag. The patient keeps tapping their foot and crossing and uncrossing their legs. The patient frequently experiences random thoughts about all the bad things that might happen to them and their loved ones. All of the reoccurring worries leave the patient feeling physically and emotionally worn out. They are experiencing frequent muscle tension, excessive sweating, and difficulties staying asleep.

# Obsessive Compulsive Disorder:

Patient F has a diagnosis of Obsessive Compulsive Disorder. Patient F has been regularly showering for prolonged periods, up to 5 hours at a time. Their skin is starting to crack and become sore, but they cannot stop themselves. The patient is preoccupied with worries that they are dirty and unclean. If they don’t shower straight away they feel panicked and as if something awful is going to happen. When they shower they must do it in a particular way so as to keep the ‘dirty bubbles’ away from the ‘clean bubbles’.

# Dissociative Identity Disorder:

Patient B has a diagnosis of Dissociative Identity Disorder. Patient B is usually a quiet and timid individual, who likes to stay home. But recently they have been acting like a completely different person. The patient is loud, outspoken, and speaks with a strong French accent. There have been hours or days where they have no recollection of what happened. The last thing they can remember before their most recent memory black-out was feeling disconnected from their body and unreal. The patient finds it difficult to recall specific moments from their childhood.

# Posttraumatic Stress Disorder:

Patient G has a diagnosis of Posttraumatic Stress Disorder. Patient G has been feeling really depressed recently. A year ago they were attacked while traveling home alone after a night out. Since the incident, they report “things have not been the same”. The patient has been irritable lately, losing their temper at the smallest of things. They have been feeling disconnected from other people, and when not angry, they feel numb inside. The patient avoids going out alone when it is dark. They try not to think about what happened to them.

# Borderline personality disorder:

Patient H has a diagnosis of Borderline Personality Disorder. Patient H frequently hurts themselves by cutting their wrists. The cuts draw blood but they never required stitches. The patient feels angry because they report that everyone they get close to keeps abandoning them. Their mood changes quite often: sometimes feeling low, sometimes anxious, sometimes irritable – but these moods tend to only last for a few hours at a time. The way they respond to situations seems to be very different to other people, and they are often told that they are overreacting.

# Antisocial personality disorder:

Patient I has a diagnosis of Antisocial Personality Disorder. Patient I has frequently been in trouble with the police. It started when they were a child and got arrested for stealing cars. When asked why they commit crimes, they respond that they were “bored and felt like it”. They do not regret any of the crimes they have committed or care if anyone got hurt. When you meet them, the patient comes across as open, engaging, and even charming. But sometimes their demeanour changes and they can be rude, dismissive and disrespectful.
