# Supplementary material for: Creating a hierarchy of mental health stigma: testing the effect of psychiatric diagnosis on stigma
Source: BJPsych Open. 2022 Sep 26;8(5):e174. doi: 10.1192/bjo.2022.578 (PMC9534883; doi:10.1192/bjo.2022.578)
Supplement: Supplementary file 1 [file bjosup.zip › S2056472422005786sup002.docx]

**Supplementary File**

| **Case Vignette Diagnosis** | ***n*** | ***M*** | ***SD*** |
| --- | --- | --- | --- |
| Schizophrenia | 578 | 2.10 | 0.64 |
| Depression | 580 | 0.97 | 0.58 |
| GAD | 574 | 1.01 | 0.58 |
| OCD | 571 | 1.10 | 0.55 |
| PTSD | 584 | 1.47 | 0.70 |
| Bipolar Type 1 | 581 | 1.57 | 0.64 |
| DID | 576 | 1.80 | 0.63 |
| BPD | 580 | 1.94 | 0.64 |
| AsPD | 575 | 2.03 | 0.64 |

**Table I.** Descriptive statistics for the Social Distance Scale in relation to the case vignette diagnosis.
